# Workforce impact of emergency department boarding

**DOI:** 10.1093/haschl/qxaf134

**Published:** 2025-07-04

**Authors:** Vicki Norton, Kraftin E Schreyer, Diane Kuhn

**Affiliations:** Florida Atlantic University Charles E. Schmidt College of Medicine, Department of Emergency Medicine, Boca Raton, FL 33431, United States; Lewis Katz School of Medicine at Temple University, Department of Emergency Medicine, Philadelphia, PA 19140, United States; Indiana University School of Medicine, Department of Emergency Medicine, Indianapolis, IN 46202, United States

**Keywords:** emergency department, boarding, burnout, violence, clinician wellness, healthcare policy

## Abstract

**Introduction:**

Emergency department (ED) boarding, or holding admitted patients in the ED due to a lack of available inpatient beds, presents a major challenge to healthcare systems. This study examines the impacts of ED boarding on clinician wellness, burnout, moral injury, and workplace violence.

**Methods:**

We distributed a survey to members of the American Academy of Emergency Medicine over 4 weeks in early 2025. It included 9 questions on experiences with boarding, demographics, and practice setting, plus an optional free-response question. Descriptive statistics were performed, and free-response questions were explored for insight and broader themes.

**Results:**

Among 195 respondents, 54.1% reported experiencing violence related to ED boarding, and 98.5% reported an impact on job satisfaction. Ninety-six respondents submitted free-text comments, which reflected 4 major themes: frustration and burnout; verbal and physical abuse; moral injury tied to profit-driven decision-making; and impacts on clinical care and patient outcomes.

**Conclusion:**

These preliminary findings that highlight additional adverse outcomes of boarding can be used to inform future policy decisions and clinical operations interventions to reduce and mitigate effects of ED boarding.

## Introduction

Emergency department (ED) boarding, defined as the practice of holding admitted patients in the ED due to a lack of available inpatient beds, is a pervasive and worsening challenge in healthcare systems worldwide.^1^ This issue contributes to downstream ED crowding, significantly compromising the capacity to provide timely care.^2^ Key contributors to ED boarding include systemic inefficiencies such as misaligned elective surgery schedules and inadequate inpatient staffing.^3^ Financial incentives for hospitals to maintain high bed occupancy rates further perpetuate the problem.^4^ Increasingly, healthcare consolidation and corporatization, including private equity investments, exacerbate these inefficiencies by prioritizing cost-cutting measures such as understaffing and resource minimization.^5,6,14^ The consequences of ED boarding extend beyond operational inefficiencies. Numerous studies have linked boarding to negative patient outcomes, including increased morbidity and mortality.^7,8^ Delayed symptom management and prolonged wait times degrade the quality of care as well as patient experience.^9^ These factors collectively highlight the urgency of addressing ED boarding as a public health crisis.

While extensive research has explored the impacts of ED boarding on patient outcomes, the effects on clinicians remain underexplored. Preliminary studies suggest that ED boarding contributes to heightened staff stress, burnout, and increased turnover rates.^10,11^ Additionally, crowding and prolonged wait times correlate with increased incidences of violence against healthcare workers, further jeopardizing workforce safety.^12,13^ The links between ED boarding and clinician well-being and safety must be further explored. To find a meaningful solution to these pervasive problems, we need better knowledge of how the systemic inefficiencies that create and perpetuate boarding directly contribute to an unsafe work environment and a demoralized workforce. A deeper understanding of these dynamics is essential to develop evidence-based interventions that prioritize clinician well-being. We sought to begin that understanding with a novel study that focuses on how front-line clinicians perceive the links between boarding and their personal well-being and safety. To our knowledge, we are the first to get the direct perspective of front-line clinicians on the interrelationship of boarding and violence. Future policies and practice interventions to preserve the workforce and clinical environment should be informed by these connections, as they will be most effective if targeted towards specific opportunities for improvement.

## Methods

The study employed a cross-sectional survey design, targeting ED physicians across urban, suburban, and rural practice settings. Participants were recruited via a professional organization, the American Academy of Emergency Medicine (AAEM), and data collection occurred over 4 weeks in early 2025. The survey instrument included items designed to capture demographic data, experiences of burnout and violence, and open-ended narratives on the personal impacts of ED boarding. We distributed the survey via email to all members of the AAEM listserv. During the 4-week data collection period, 1 reminder email was sent regarding the survey. A copy of the survey is included in Appendix A.

## Results

There were 195 physician respondents from within AAEM and the AAEM Resident and Student Association. The characteristics of survey respondents are shown in Table 1.

**Table 1.** Characteristics of survey respondents.

	Mean (SD)
Age	48.1 (11.4)
Years out of residency	17.1 (11.1)
	*n* (percentage of total)
Gender	
Male	149 (76.0)
Female	41 (20.9)
Nonbinary/not provided	6 (3.1)
Race	
White	162 (82.7)
Asian	9 (4.6)
Black or African American	3 (1.5)
Other, multiracial, or unknown	22 (11.2)
Ethnicity	
Not Hispanic or Latinx	163 (83.2)
Hispanic or Latinx	25 (12.8)
Other or unknown	8 (4.1)
Primary practice setting	
Urban	93 (47.4)
Suburban	79 (40.3)
Rural	24 (12.2)

Of the respondents, all but 3 (192/195, 98.5%) reported that ED boarding has impacted their job satisfaction, and 169/195 (86.7%) reported that boarding had led them to experience burnout or moral injury. In addition, 105 respondents (53.8%) reported having personally experienced violence because of boarding or downstream effects such as ED crowding, and 157 (80.5%) reported having experienced violence toward another member of the ED staff. Ninety-six respondents elected to provide free-response comments regarding experiences with boarding. Comments were broadly separated into 4 categories: (1) frustration and burnout, (2) verbal and physical abuse, (3) moral injury and perception that addressing ED boarding is not a priority given its use as a tool to maximize profitability, and (4) effect of boarding on clinical care and outcomes. Representative comments on these topics are provided in Table 2. A total of 29 comments were either related to other topics or, more commonly, general remarks such as “too many stories to count” or “everyday issue”.

**Table 2.** Topics and comments related to ED boarding.

Topic	Representative comments
ED boarding leads to patient and staff frustration, burnout, and consideration for leaving the profession	“Boarding impacts patient wellness, safety, staff safety and wellness…” “This is one of the two top issues that negatively impact my satisfaction with EM, has me looking for an early exit, and gives me pause in recommending the profession to other aspiring physicians. When your workplace is akin to a warzone MASH unit…it is difficult to remain focused on the silver lining of this profession—that we care for all comers, 24/7, 365…and do so with an expertise and breadth of knowledge that is unmatched by any other specialty.” “Many of our more experienced nurses have resigned because they did not sign up to provide inpatient care in the ED.” “Moral injury of watching suffering and not having the tools and space to help, nurses leaving the profession because they’re being asked to participate in inpatient nursing and take care of ED patients.” “Having to start EVERY patient encounter with ‘I'm so sorry you had to wait so long…’ definitely wears on you.” “Psych patients that stay in ED for many days is a disaster and demoralizing for both the patient and staff.” “Nurses are leaving, physician assistants and nurse practitioners are leaving, and unhappiness among staff is at an all time high, even when the Boomer generation is entering their time of greatest need.” “I have several unflappable colleagues who are at their breaking point. We use secure chat and one person had 100 secure chats from the nurses about boarded patients in his first few hours of work. Bad for all.” “I left my last job because the poor quality of care for boarding patients made it untenable.” “Frustrated patients. Frustrated staff.”
Verbal and/or physical abuse of staff	“…constant anger and disdain from patients and family who are understandably scared and frustrated about the delays in their care, which commonly manifests as verbal abuse, but have experienced threats of physical abuse as well more than once.” “Just today, I was approached by our triage nurse (a very experienced nurse) who came back into the ED to report that she felt unsafe (and was worried for the patients in the waiting room) due to the level of unrest in our ED waiting room due to long wait times- we were boarding 30 admissions and had more than 30 people waiting in the waiting room at the time.” “Repeatedly getting the brunt of referral physicians yelling at ED physicians for their ill patients in the waiting room for hours…” “As a clinician but also a medical director, ED boarding is a huge quality and safety issue. Left without being seen rates go up (and not all of those are low-risk complaints), violence increases…” “Patients who start off relatively pleased when you see them fast, turn on you after they wait hours and hours in the waiting room thereafter.”
Moral injury/perception that hospital administrators are focused on profitability at the expense of emergency care and serving as a safety net	“In the recent weeks we have boarded over 80 inpatients, and sometimes surpassing 90, in our emergency department. This causes severe moral injury to staff not having the resources to treat sick patients that continue to come through the door.” “The real moral injury is not due to the volume and/or boarding itself. It's the response. As experts trained in managing disasters, we are well educated on concepts of surge capacity and capability. We have slowly become accustomed to the idea that our healthcare organizations have removed surge capacity and capability from our departments and hospitals, in favor of efficiency and maximizing profitability. How would the public respond if we drew down firefighting resources in favor of economic optimization? Eliminating resources so that fire crews go from one call to the next, continuously operating at maximal ‘efficiency’, but incapable of reaching some fires in a timely fashion and unable to reach others when calls overlap? That is what we have done with our industry. Healthcare in this country is headed for a reckoning, it's inevitable. I just worry about what will occur in the time it takes us to get there.” “I personally feel the hospital administration is not making this a priority. There does not appear to be a push for early discharges on the floor/ward services. It almost feels that we are left out to dry. Some of the worst boarding that I have seen in 25 years.” “We are the basement dwellers—Boarding and reimbursement cuts underscore that we are the dumping grounds of hospital administrators and public/private payers alike.” “Administrators hound us to compromise care or participate in initiatives to “fix” boarding—which are rarely helpful because the ED isn’t the cause of the problem.” “At our hospital, the boarding issue is primarily driven by lack of inpatient beds, due to difficulty discharging patients to subacute rehab and due to inpatient surgery volumes. There is seemingly a prioritization of surgical patients over emergency department patients.” “Excessive boarders represent a critical failure to deliver safe care to our most vulnerable patients and showcase neglect of duty from senior administration to uphold ethical standards. This situation also fuels the ongoing burnout experienced by ED staff and must be urgently addressed.” “Good management distributes stress across the organization. In medicine, admitted patients are stacked in the emergency department as it is the easiest solution. Why make ten inpatient units unhappy by doing 5% more work when you can just dump everything on the emergency department and overload it by 50% or more. If the emergency physician complains they are fired. This is facilitated by hiring physician contract management groups that offer a ‘fire at will’ clause in the parent agreement whereupon the CMG can serve as work around to whistleblower laws at the behest of unscrupulous hospital administrators—all for the right to extract clinical revenue from the emergency physician—in essence the emergency physician pays a tax that funds their own subjugation and mistreatment of their patients.” “Work in a seasonal community and every year we have the same expected issues and administration offers the same minimal efforts at solutions.” “I've had to respond to questions from administration regarding delays in metrics while my calls for innovation and hospital-wide efforts to address boarding go unanswered.”
ED boarding leads to practicing medicine in the hallways and waiting room, compromising the quality of care and patient outcomes	“Unfortunately boarding has caused some unsafe practices in our ED. I have had patients having an active MI [myocardial infarction]]or with severe hyperkalemia that were not on a telemonitor. We have to do a game of musical chairs to get them onto a monitored bed. Feels like a matter of time before one of these patients goes into cardiac arrest sitting in a chair in the hallway or waiting room because we have 22 admit holds in an ED that was designed to only accommodate 20 beds.” “I see close to 3/4 of my patients in chairs or ambulance gurneys because the few beds we have that aren't used for boarders are reserved for truly critical patients.” “Patient[s] put in hallway without nurse - have witnessed deaths as a result…” “ED boarding creates a situation where we are examining people in chairs, hallways, lobbies, and just about anywhere. This is far from ideal and leads to poor encounters for the [physician or other clinician] and patient.” “Because the department has been full with boarded patients, even though we have created chairs in the hall near triage to try to see patients, we cannot see patients as soon as we want to. These hours-long waits have resulted in patients with critical illnesses waiting hours to be seen, and we often cannot do a full exam while the patient sits in a chair or ask a full history because of the proximity of other people. This is not how we want to take care of our community.” “I have seen multiple patients over the years code in the waiting room due to lack of ED beds from boarding.” “…missed strokes, STEMIs, sepsis from patient waiting…” “‘Waiting Room Medicine.’ Working patients up and dispositioning a patient entirely from the waiting room. I once diagnosed a patient with an acute ischemic limb in the waiting room, had the CTa while still in the waiting room, and had to start her heparin drip in the waiting room.” “It has caused direct patient harm. Coding patients in [the] waiting room. Evaluating patients in hallways where privacy is impacted. Inability to perform adequate physical examinations and provide standard of care management.” “2 times patients have died in lobby.” “We had a patient code in the waiting room who arrived with CP [chest pain] and had no monitored bed to place him… it could have been avoided with monitoring.” “We have had a patient with a STEMI sit in the waiting room for hours and eventually die on the cath lab table due to significant waiting room times directly related to the 20 boarders in our 19 bed ED.” “I can report consistent delays in evaluation and diagnosis, poor patient experiences, increase in patients leaving before being seen or before treatment could be completed. I've seen boarding cause delays in consults, treatments, and management for patients boarded in the ED that would not see those delays if they were admitted on the floor.” “We have had patients die from hypoxic brain injury after their oxygen tank runs out of oxygen, as we didn't have enough oxygen monitors. We have had patients die in the waiting room, who should have been in a monitored cardiac bed. We have performed rectal exams in the hallway, due to lack of alternative options. We have missed necrotizing infections due to septic patients who are fully clothed in a hallway stretcher and unable to perform a skin exam. These are not rare or once-off stories, but monthly occurrences. I work in an ER chartered for 44 ER beds and yet we exclusively operate with only 6-10 functional ER beds then put people in 8 recliners for fast track and 15 in hallways. The ER is the largest inpatient unit in the hospital at all times, with a minimum 25+ boarding patients at any given time (as high as 60).”
Impacts on education and training	“It has negative consequences on the education of our residents. Besides decreasing the volume of cases they can see in a shift, it may affect the acuity of the patients they see. They also witness the frustration of their attendings, which can have under-appreciated consequences in their development.” “Compromises care and the education of the next generation of EM physicians. The doctors in training cannot see new patients when the beds are tied up with boarders.” “The lack of total ED volume has negatively impacted both our community and our residency training.”

Abbreviations: CTa, computed tomography angiogram; ED, emergency department; ER, emergency room; MASH, Mobile Army Surgical Hospital; STEMI, ST-elevation myocardial infarction.

## Discussion

The findings of this study underscore the detrimental impacts of ED boarding on both patient and clinician wellness and safety in a novel way, through the perspective of the front-line clinicians who experience the systemic inefficiencies of boarding on a routine basis. As has been aforenoted, survey respondents frequently described scenarios where critical patients suffered harm due to prolonged ED stays, lack of monitoring, and overcrowded conditions, with multiple individuals commenting on cardiac arrests and patient deaths in the waiting room. In addition to highlighting previously noted adverse patient outcomes that result from boarding, respondents also expressed concerns that emergency care provided in the waiting room and hallways was of a lower quality than patients deserved.

On the clinician side, frustration and burnout were commonly reported. Consistent reports of staff leaving the profession due to untenable working conditions indicate that continued inaction on ED boarding may exacerbate workforce shortages in emergency medicine, both by impacting current clinicians and trainees.

Additionally, the high prevalence of verbal and physical abuse highlights the urgency of enhanced security measures and de-escalation training to protect frontline workers. Over half of the respondents in this study reported experiencing an incident of violence as a result of boarding. Notably, 80.6% of respondents reported witnessing violence toward a colleague. These both indicate a pervasive and alarming trend. The prevalence of burnout among ED staff aligns with prior research linking occupational stress to systemic inefficiencies and resource constraints.^10^ The high incidence of violence reported by respondents echoes concerns raised by prior studies, emphasizing the need for immediate action to protect healthcare workers.^12^

A recurring theme in the survey responses was the inherent tension between financial incentives and the provision of safe patient care, with respondents expressing concern that profit-driven decision-making often compromises clinical standards. Importantly, respondents noted that the lack of willingness of administrators to hear those concerns also has a marked impact on clinician burnout. Healthcare consolidation and corporatization, including private equity investments, have been an important contributing factor to this environment. Although most states have corporate practice of medicine (CPOM) laws restricting nonphysician entities such as corporations from practicing medicine or employing physicians, their enforcement varies significantly.^15^ In states with weak or unenforced CPOM laws, corporate entities can influence physician staffing, resource allocation, and operational policies in EDs, exacerbating ED boarding issues.^16^ As a result of the lack of enforcement, some states have sought to strengthen their CPOM laws. For example, in 2025, California introduced legislation to ban corporate investors from influencing medical practices, while Oregon and Vermont have proposed even stricter measures.^17^

Overall, our findings underscore the urgent need for systemic reforms to mitigate the effects of ED boarding. Although the sample was targeted, respondents represent clinicians working on the front line, facing these important issues on every shift. Addressing the root causes, such as inefficient bed management, inadequate inpatient staffing, hospital policies that prioritize elective procedures over emergency admissions, and corporatization, is essential to ensuring both patient and clinician well-being. Specific improvements should be made to ensure the availability of resources to provide high-quality care and to ensure a safe work environment.

Our study has several limitations. First, we surveyed members of AAEM, which may not be representative of emergency physicians as a whole. In addition, respondents may have been more inclined to participate in a survey on ED boarding if it was a concern of theirs, meaning that the percentage of respondents reporting issues related to ED boarding and workforce violence may overrepresent the prevalence in the population of emergency physicians. In addition, this is an exploratory study, looking at brief free-response text rather than qualitative interviews. Nonetheless, we believe that it provides important insight into how ED boarding may be impacting our workforce.

## Policy proposal

To alleviate ED boarding, hospitals must implement dynamic bed allocation strategies, including real-time capacity monitoring and prioritization of ED admissions. Initiatives such as staggered elective surgery schedules could mitigate peak demand pressures.^3^ Investments in inpatient staffing, including flexible staffing pools, are critical to reducing boarding times and alleviating ED congestion.^11^ Requiring hospitals to provide adequate inpatient care for inpatients boarding in the ED might then improve care of ED patients who would benefit from the staff resources they were intended to have. Policymakers and healthcare leaders should also reevaluate reimbursement models that incentivize high occupancy rates and cost-cutting practices, exacerbated by the corporatization of medicine, at the expense of efficient care delivery.^4,6,14^ Making boarding a reportable quality metric would also distribute the burden of alleviating boarding to the administrators outside of the ED. This contrasts with current metrics, such as LWBS, which is often thought of as an ED-centric metric, even though it is reflective of upstream congestion.

While the primary policy objective should be the reduction of ED boarding itself, institutions can also take steps to mitigate its negative impact on clinicians. Health systems must prioritize clinician wellness by offering accessible mental health resources, peer support programs, and regular debriefings to address the psychological toll of ED boarding. Hospitals should also adopt comprehensive violence prevention programs, including staff training, de-escalation protocols, and enhanced security measures. Legislative advocacy for stronger protections for healthcare workers is also imperative.^12^ By implementing these strategies, healthcare systems can mitigate the adverse effects of ED boarding on both patients and clinicians, fostering a safer and more sustainable work environment.

## Conclusion

This study highlights the challenges posed by ED boarding, emphasizing its detrimental effects on clinician wellness, patient safety, and healthcare system efficiency. The results demonstrate that ED boarding not only leads to burnout and moral injury among clinicians but also increases violence in the workplace and negatively impacts clinical outcomes. Implementing dynamic bed allocation strategies, increasing inpatient staffing, strengthening CPOM laws, and incorporating comprehensive anti-violence strategies are critical steps in reducing the burden and impact of boarding. Future research should focus on long-term policy outcomes and innovative care models to create a more sustainable and equitable emergency care system for both patients and clinicians.

## Supplementary Material

qxaf134_Supplementary_Data
