# Supplementary material for: Workforce impact of emergency department boarding
Source: Health Aff Sch. 2025 Jul 4;3(8):qxaf134. doi: 10.1093/haschl/qxaf134 (PMC12342924; doi:10.1093/haschl/qxaf134)
Supplement: qxaf134_Supplementary_Data [file qxaf134_supplementary_data.zip › Appendix A.docx]

**Appendix A**

Survey on ED Boarding Impact on Wellness and Safety

AAEM is looking for your input on the impact of boarding admitted patients in the Emergency Department for the Agency for Healthcare Research and Quality (AHRQ). ED Boarding is defined by the Joint Commission (JCAHO) as the time between the decision to admit a patient and their physical departure from the ED, and it is recommended this time not exceed four hours. Currently, there are no metrics or quality measures enforcing limits on boarding time. AAEM is trying to gather data on the effects of ED Boarding on workforce wellness and safety.

As an AAEM member, you are being asked to participate in this important, potentially policy-changing, survey. This should only take about 2 minutes of your time. Your participation is completely voluntary, but much appreciated. The survey will be available until Friday, January 31st.

• Has the boarding of admitted patients in your emergency department negatively impacted your job satisfaction? (Yes/No)

• Has boarding caused you any feelings of “burnout” or moral injury? (Yes/No)

• Have you personally experienced any violence from patients as a result of boarding or its downstream effects (ED crowding, increased wait times, etc.)? (Yes/No)

• Have you experienced any violence towards your staff and/or colleagues as a result of boarding or its downstream effects (ED crowding, increased wait times, etc.)? (Yes/No)

• Do you have a story regarding ED boarding and its impact on you or your colleagues? (open-ended)

• I am a: (medical student, physician)

• Age

• Gender (Male, Female, Non-binary, Prefer not to disclose, Prefer to self-describe, Unknown)

• Race/Ethnicity: Choose all that apply (American Indian or Alaska Native, Asian, Black or African American, Native Hawaiian or Other Pacific Islander/White, Unknown, Hispanic or Latino/x, Other:(fill in blank))

• Primary Practice Setting: Urban, Suburban, Rural

• Number of years in Practice
